# Enhancing human gut health: Global innovations in dysbiosis management

**DOI:** 10.1002/imt2.70028

**Published:** 2025-04-13

**Authors:** Reda El Boukhari, Maima Matin, Latifa Bouissane, Michał Ławiński, Oleh Lushchak, Rajeev K. Singla, Michel‐Edwar Mickael, Jordi Mayneris‐Perxachs, Maria Eleni Grafakou, Shuhua Xu, Bowen Liu, Jiayi Guan, Andrzej Półtorak, Arkadiusz Szpicer, Agnieszka Wierzbicka, Nikolay T. Tzvetkov, Maciej Banach, Jarosław Olav Horbańczuk, Artur Jóźwik, Marco Cascella, Bairong Shen, Vasil Radoslavov Pirgozliev, Dongdong Wang, Olena Litvinova, Olga Adamska, Agnieszka Kamińska, Marcin Łapiński, Artur Stolarczyk, Ioana Berindan‐Neagoe, Luigi Milella, Andy Wai Kan Yeung, Prashanth Suravajhala, Anupam Bishayee, Ronan Lordan, Laszlo Barna Iantovics, Ricardo Lagoa, Monika Michalczuk, Jivko Stoyanov, A. Douglas Kinghorn, Banaz Jalil, Wolfram Weckwerth, Bey Hing Goh, Meng‐Yao Li, Gyaneshwer Chaubey, Gian Luigi Russo, Sara Frazzini, Luciana Rossi, Maurizio Battino, Wei Jia, Qi Su, Xiaoqiang Ma, Judith M. Rollinger, Simon K.‐M. R. Rittmann, Helen Sheridan, John J. Walsh, Gérard Lizard, Tomasz M. Karpiński, Ana Sanches Silva, Jakub Piwowarski, Liwei Xie, Tai‐Ping Fan, Francesca Giampieri, Adil El Midaoui, Ka‐Hing Wong, Ren‐You Gan, Ahmed Fatimi, Atanas G. Atanasov

**Affiliations:** ^1^ Chemical Science and Engineering Research Team (ERSIC), Department of Chemistry, Polydisciplinary Faculty of Beni Mellal (FPBM) Sultan Moulay Slimane University (USMS) Beni Mellal Morocco; ^2^ Institute of Genetics and Animal Biotechnology of the Polish Academy of Sciences Jastrzębiec Poland; ^3^ Molecular Chemistry, Materials and Catalysis Laboratory, Faculty of Sciences and Technologies Sultan Moulay Slimane University Beni Mellal Morocco; ^4^ Department of General, Gastroenterologic and Oncologic Surgery Medical University of Warsaw Warsaw Poland; ^5^ Department of Biochemistry and Biotechnology Vasyl Stefanyk Precarpathian National University Ivano‐Frankivsk Ukraine; ^6^ Research and Development University Ivano‐Frankivsk Ukraine; ^7^ Department of Pharmacy and Institutes for Systems Genetics, Center for High Altitude Medicine, Frontiers Science Center for Disease‐related Molecular Network, West China Hospital Sichuan University Chengdu Sichuan China; ^8^ School of Pharmaceutical Sciences Lovely Professional University Phagwara Punjab India; ^9^ Department of Diabetes, Endocrinology and Nutrition Dr. Josep Trueta University Hospital Girona Spain; ^10^ CIBER Fisiopatología de la Obesidad y Nutrición (CIBERobn) Madrid Spain; ^11^ Integrative Systems Medicine and Biology Group, Girona Biomedical Research Institute (IDIBGI‐CERCA) Parc Hospitalari Martí i Julià Salt Spain; ^12^ Chair of Pharmaceutical Biology, Faculty of Pharmacy and Chemistry University of Regensburg Germany; ^13^ Center for Evolutionary Biology, School of Life Sciences Fudan University Shanghai China; ^14^ Human Phenome Institute, Zhangjiang Fudan International Innovation Center Fudan University Shanghai China; ^15^ School of Agriculture Yunnan University Kunming China; ^16^ Henan Institute of Medical and Pharmaceutical Sciences Zhengzhou University Zhengzhou China; ^17^ Department of Technique and Food Development, Institute of Human Nutrition Sciences Warsaw University of Life Sciences Warsaw Poland; ^18^ Department of Biochemical Pharmacology and Drug Design, Institute of Molecular Biology “Roumen Tsanev” Bulgarian Academy of Sciences Sofia Bulgaria; ^19^ Faculty of Medicine The John Paul II Catholic University of Lublin (KUL) Lublin Poland; ^20^ Department of Cardiology and Adult Congenital Heart Diseases Polish Mother's Memorial Hospital Research Institute (PMMHRI) Lodz Poland; ^21^ Department of Preventive Cardiology and Lipidology Medical University of Lodz (MUL) Lodz Poland; ^22^ Ciccarone Center for the Prevention of Cardiovascular Disease Johns Hopkins University School of Medicine Baltimore Maryland USA; ^23^ Anesthesia and Pain Medicine, Department of Medicine, Surgery and Dentistry “Scuola MedicaSalernitana” University of Salerno Baronissi Italy; ^24^ Department of Critical Care Medicine and Institutes for Systems Genetics Frontiers Science Center for Disease‐Related Molecular Network, West China Hospital,Sichuan University Chengdu Sichuan China; ^25^ Center for High Altitude Medicine, West China Hospital Sichuan University Chengdu Sichuan China; ^26^ National Institute of Poultry Husbandry Harper Adams University Newport UK; ^27^ Centre for Metabolism, Obesity and Diabetes Research McMaster University Hamilton Ontario Canada; ^28^ Division of Endocrinology and Metabolism, Department of Medicine McMaster University Hamilton Ontario Canada; ^29^ National University of Pharmacy of the Ministry of Health of Ukraine Kharkiv Ukraine; ^30^ Ludwig Boltzmann Institute Digital Health and Patient Safety Medical University of Vienna Vienna Austria; ^31^ Faculty of Medicine Collegium Medicum Cardinal Stefan Wyszyński University in Warsaw Warsaw Poland; ^32^ Orthopaedic and Rehabilitation Department Medical University of Warsaw Warsaw Poland; ^33^ Department of Genomics MEDFUTURE ‐ Institute for Biomedical Research“Iuliu Hațieganu” University of Medicine and Pharmacy No. 23 Cluj‐Napoca Romania; ^34^ Department of Health Sciences University of Basilicata Potenza Italy; ^35^ Oral and Maxillofacial Radiology, Applied Oral Sciences and Community Dental Care, Faculty of Dentistry The University of Hong Kong Pokfulam Hong Kong SAR; ^36^ Amrita School of Biotechnology Amrita Viswa Vidyapeetham Clappana Kerala India; ^37^ Department of Biosciences Manipal University Jaipur, Dehmi Kala Jaipur Rajasthan India; ^38^ Department of Pharmacology College of Osteopathic Medicine, Lake Erie College of Osteopathic Medicine Bradenton Florida USA; ^39^ The Institute for Translational Medicine and Therapeutics, Perelman School of Medicine University of Pennsylvania Philadelphia Pennsylvania USA; ^40^ Department of Electrical Engineering and Information Technology George Emil Palade University of Medicine, Pharmacy, Science, and Technology of Targu Mures Targu Mures Romania; ^41^ ESTG‐Polytechnic Institute of Leiria Morro do Lena‐Alto do Vieiro Leiria Portugal; ^42^ LSRE‐LCM‐Associate Laboratory in Chemical Engineering University of Porto Porto Portugal; ^43^ Department of Animal Breeding, Institute of Animal Sciences Warsaw University of Life Sciences ‐ SGGW Warsaw Poland; ^44^ Swiss Paraplegic Research Nottwil Switzerland; ^45^ Institute of Social and Preventive Medicine (ISPM) University of Bern Bern Switzerland; ^46^ College of Pharmacy Ohio State University Columbus Ohio USA; ^47^ Pharmacognosy and Phytotherapy UCL School of Pharmacy London UK; ^48^ Molecular Systems Biology Lab (MOSYS), Department of Functional and Evolutionary Ecology University of Vienna Vienna Austria; ^49^ Vienna Metabolomics Center (VIME) University of Vienna Vienna Austria; ^50^ Sunway Biofunctional Molecules Discovery Centre (SBMDC) School of Medical and Life Sciences Subang Jaya Malaysia; ^51^ Biofunctional Molecule Exploratory (BMEX) Research Group, School of Pharmacy Monash University Malaysia Subang Jaya Malaysia; ^52^ Faculty of Health, Australian Research Centre in Complementary and Integrative Medicine University of Technology Sydney Ultimo New South Wales Australia; ^53^ State Key Laboratory of Systems Medicine for Cancer, Shanghai Cancer Institute, Renji Hospital Shanghai Jiao Tong University School of Medicine Shanghai China; ^54^ Department of Biliary‐Pancreatic Surgery, Renji Hospital Shanghai Jiao Tong University School of Medicine Shanghai China; ^55^ Cytogenetics Laboratory, Department of Zoology Banaras Hindu University Varanasi Uttar Pradesh India; ^56^ National Research Council Institute of Food Sciences Avellino Italy; ^57^ Department of Veterinary Medicine and Animal Science (DIVAS) University of Milan Lodi Italy; ^58^ Department of Clinical Sciences Polytechnic University of Marche Ancona Italy; ^59^ Joint Laboratory on Food Science, Nutrition, and Intelligent Processing of Foods Polytechnic University of Marche (Italy), Universidad Europea del Atlántico (Spain), and Jiangsu University (China) Ancona Italy; ^60^ International Joint Research, Laboratory of Intelligent Agriculture and Agri‐Products Processing Jiangsu University Zhenjiang China; ^61^ Department of Pharmacology and Pharmacy The University of Hong Kong Pokfulam Hong Kong SAR; ^62^ Microbiota I‐Center Shatin Hong Kong SAR; ^63^ Department of Medicine and Therapeutics The Chinese University of Hong Kong Shatin Hong Kong SAR; ^64^ Department of Food Science and Technology, School of Agriculture and Biology Shanghai Jiao Tong University Shanghai China; ^65^ Division of Pharmacognosy, Department of Pharmaceutical Sciences, Faculty of Life Sciences University of Vienna Vienna Austria; ^66^ Archaea Physiology & Biotechnology Group, Department of Functional and Evolutionary Ecology University of Vienna Vienna Austria; ^67^ The NatPro Centre & School of Pharmacy and Pharmaceutical Sciences Trinity College Dublin Dublin Ireland; ^68^ Université Bourgogne Europe/INSERM, 21000 Dijon and PHYNOHA Consulting Fontaine‐lès‐Dijon France; ^69^ Department of Medical Microbiology Poznań University of Medical Sciences Poznań Poland; ^70^ University of Coimbra, Faculty of Pharmacy, Polo III, Azinhaga de Santa Comba Coimbra Portugal; ^71^ Centre for Animal Science Studies (CECA), ICETA University of Porto Porto Portugal; ^72^ Microbiota Lab, Department of Pharmaceutical Microbiology and Bioanalysis Medical University of Warsaw Warsaw Poland; ^73^ State Key Laboratory of Applied Microbiology Southern China, Guangdong Provincial Key Laboratory of Microbial Culture Collection and Application, Guangdong Open Laboratory of Applied Microbiology Institute of Microbiology, Guangdong Academy of Sciences Guangzhou China; ^74^ School of Life & Health Sciences Fuyao University of Science & Technology Fuzhou Fujian China; ^75^ Research Group on Food, Nutritional Biochemistry and Health Universidad Europea del Atlántico Santander Spain; ^76^ International Research Center for Food Nutrition and Safety Jiangsu University Zhenjiang China; ^77^ Faculty of Sciences and Techniques Errachidia, Moulay Ismail University of Meknes Meknes Morocco; ^78^ Department of Pharmacology and Physiology, Faculty of Medicine University of Montreal Montreal Quebec Canada; ^79^ Research Institute for Future Food The Hong Kong Polytechnic University Hung Hom Hong Kong SAR; ^80^ Department of Food Science and Nutrition The Hong Kong Polytechnic University Hung Hom Hong Kong SAR; ^81^ Laboratory of Natural Products and Medicinal Chemistry (LNPMC), Center for Global Health Research, Saveetha Medical College and Hospital Saveetha Institute of Medical and Technical Sciences (SIMATS) Thandalam Chennai India

## Abstract

The microbiota, comprising all the microorganisms within the body, plays a critical role in maintaining good health. Dysbiosis represents a condition resulting from an imbalance or alteration of the microbiota. This study comprehensively investigates the patent literature on dysbiosis over the past 20 years.

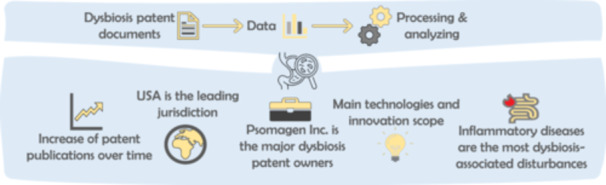

To the Editor,

The gut microbiota represents all the microorganisms, mainly bacteria, that coexist in the digestive system and are mostly beneficial. In adulthood, the gut microbiota composition, which varies from person to person, generally remains stable with a particular density in each part of the digestive system. However, a microbial community imbalance within the body, particularly in the gut, is sometimes observed, causing dysbiosis characterized by reduced microbial diversity, loss of beneficial bacteria, and increased pathogenic microorganisms [1, 2].

The term “dysbiosis” has been used since at least 1897, as indicated by the Google Books Ngram Viewer (https://books.google.com/ngrams; accessed on December 17, 2024). Furthermore, the term dysbiosis was first explicitly used in the 1949 book by Hutyra et al. [3], and gained prominence in the 1960s and 1970s. During this period, researchers such as Hans Haenel emphasized the importance of quantifying dysbiosis and its counterpart, eubiosis, in relation to disease. However, modern research has not extensively adopted this quantitative approach [4].

Several studies have revealed the causes of dysbiosis, including the excessive use of antibiotics [2], certain classes of nonantibiotic prescription medications (e.g., chemotherapy), a high‐fat, low‐fiber diet [5], sedentary lifestyle factors, circadian disruption and insomnia, smoking [2], and early factors such as the mode of fetus delivery and breastfeeding [1]. Common symptoms of dysbiosis are gastrointestinal disturbances (e.g., abdominal pain, distension, and diarrhea) [6]. Other symptoms may include fatigue, mental confusion, skin rashes, food intolerances, and increased susceptibility to infections. Nevertheless, several diseases and health conditions, such as cancers, inflammatory, metabolic or autoimmune conditions, cardiovascular diseases, and skin conditions, also mental disorders and neurological diseases, can be associated with dysbiosis [2, 7, 8, 9, 10, 11]. Emerging research establishes the pathogenesis of microbiota dysbiosis (mainly the bacterial component) in disease development and progression, primarily through modulation of host immune response, induction of chronic inflammation, and impaired intestinal permeability, as well as abnormal microbial metabolite profiles like trimethylamine, genotoxic compounds, short‐chain fatty acids (SCFAs), bile acids, etc. [12, 13]. Understanding these mechanisms has allowed for the development of microbiota‐based treatments and innovative solutions with the aim to restore microbiome balance and mitigate associated health risks. Emerging treatments aim to leverage personalized approaches, including tailored probiotics, nutritherapy, functional foods, microbiome‐modulating dietary recommendations, and advanced microbiota transplantation techniques. The therapeutic strategies discussed for dysbiosis include the restoration of the microbial balance through dietary action and the use of probiotics, prebiotics, symbiotics, and postbiotics [2, 6, 7, 14]. The control of antibiotic‐resistant bacteria has also been studied [15]. Adverse effects of microbiome interventions are often overlooked, yet they are crucial for informed risk‐benefit assessments. Both acute and long‐term risks have been described so far, including alterations in the microbiota composition or function, invasive infections, antibiotic resistance, and chronic health risks such as autoimmune disorders and human‐to‐human pathogen transfer [16, 17, 18].

The above‐mentioned strategies underscore the intersection of science and technology in addressing microbiome‐related disorders by developing innovative solutions and inventions. Innovations are by definition novel, nonobvious and industrially applicable technical solutions, and their role is particularly evident in the patent landscape, where numerous inventions are driving progress in microbiome therapeutics.

Patents are legal documents allowing the granting of exclusivity on original methods and formulations. The patent literature, consisting of both pending and granted patents, is a valuable source for exploring the state of the art and trends around a subject of study. The analysis of patent documents can focus on the evolution of inventive research over time, as well as stakeholders such as inventors, applicants, and patent owners. The technologies covered by patents can be identified from a qualitative study or through the patent classification codes assigned to each patent when examined by intellectual property (IP) or patent offices.

The perceived high importance of dysbiosis contraction was demonstrated by a recent analysis of the overall functional food patent landscape, which revealed that probiotics and prebiotics represented a significant theme [19]. Examples of patents in this domain include engineered probiotics designed to target specific pathogens (WO2020/139852A1), prebiotic formulations optimized for gut health (WO2018/023003A1), and diagnostic tools capable of identifying dysbiosis‐related markers (US2018/0267037A1 and CN110097928B). Additionally, advancements in biotechnological platforms for microbiome analysis can facilitate the development of precision medicine approaches (US2023/0245733A1). These patents protect novel solutions and highlight the increasing commercial interest in translating microbiome science into practical healthcare applications.

Our present research aims to characterize and assess the innovation around dysbiosis through a comprehensive analysis of the patent literature, collected from *The Lens* patent database (www.lens.org). This study provides researchers with an overview of the patent landscape surrounding dysbiosis.

## RESULTS

**Figure 1:**
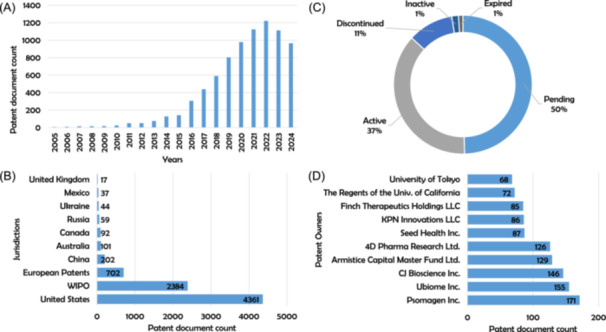
The global patent landscape of inventions related to dysbiosis. (A) Evolution of the dysbiosis patent publication since 2005 (the year 2024 data are limited to October 28). (B) Current legal status of dysbiosis patent documents. (C) Top 10 dysbiosis patent documents jurisdictions. (D) Top 10 dysbiosis patent documents owners.

To collect the major body of patents on dysbiosis, we chose to use the specialized database *The Lens*, containing 159,589,594 patent documents as of October 28, 2024. We used a query with the keyword “dysbiosis” in all patent fields, covering the period of the last 20 years from January 1, 2005, to October 28, 2024. The search returned 8097 patent documents (https://doi.org/10.5281/zenodo.14984713). The growing trend of patent filings is apparent. In 2005, only six documents were published, while the year 2022 saw a maximum number of publications with 1222 documents (Figure 1A), and 37% of the published documents are still active (Figure 1B). The United States is the jurisdiction where the most documents have been published, with 4361 patent documents (Figure 1C), and the American company Psomagen Inc. has the highest number (171 documents) of patents related to dysbiosis (Figure 1D).

**Figure 2:**
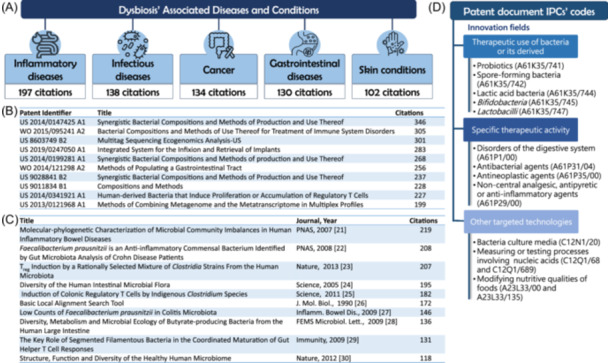
Dysbiosis‐related data based on relevant diseases, most cited patents and publications, and innovation fields. (A) The most dysbiosis‐associated diseases. (B) The top 10 most cited dysbiosis patent documents. (C) The top 10 scientific publications that are most cited in the dysbiosis patent documents. (D) Innovation fields based on the dysbiosis patent document IPC codes.

Analysis of the titles of collected patent documents shows that dysbiosis is associated with various diseases. We carried out this analysis on the titles of the documents grouped into simple families to avoid multiplying the citation of the same work. The list of titles for all 8097 documents was downloaded and processed by Python (Python Software Foundation, Wilmington, DE, United States). The most cited diseases (Figure 2A) are inflammatory diseases (197 citations), followed by infectious diseases (138 citations). The top 10 most cited patent documents related to dysbiosis (Figure 2B) had citations between 199 times (US2013/0121968A1) and 346 times (US2014/0147425A1). The top 10 most cited scientific publications in dysbiosis patent documents (Figure 2C) have received between 118 citations for the article entitled “*Structure, function, and diversity of the healthy human microbiome*” and 219 citations for the article entitled “*Molecular‐phylogenetic characterization of microbial community imbalances in human inflammatory bowel diseases.*” Based on the International Patent Classification (IPC) Portal, which is administered by the World Intellectual Property Organization (WIPO) and available at https://ipcpub.wipo.int, the analysis of the IPC codes assigned to the collected patent documents reveals that the use of bacteria is an important technological axis of innovation in the field of dysbiosis. It is represented by the general code A61K35/74 assigned to 1483 documents. The specific therapeutic activity targeted by the invention is the second technological field revealed with this analysis (Figure 2D).

## DISCUSSION

This analysis covers 8097 patent documents related to dysbiosis. These documents cover the last 20 years and come from the database specializing in patent literature, *The Lens*. Several points are of high importance and are detailed in the following discussion. A period of 20 years has been chosen as the analysis period because it encompasses more than 99% of all publications concerning dysbiosis. The trend of publications represented by the graph in Figure 1A is increasing. Innovation was very limited from the mid‐2000s to the early 2010s (between 6 and 75 documents published from 2005 to 2013). It was only in 2014 that the number of published documents exceeded 100 for the first time, with 128 patent documents published. Since then, dysbiosis has sparked a growing interest to reach a thousand publications per year from the beginning of the 2020s, with a peak recorded in 2022 when 1222 patent documents were published, including 281 granted patents. Furthermore, the distribution of publications over time within the jurisdictions with the highest numbers of publications shows that innovation concerning dysbiosis has become a notable trend since the early 2010s. Almost at the same time, the publication of patent documents in the United States, under the global Patent Cooperation Treaty (PCT) via WIPO, and in the European region, has seen a continuous increase, always dominated by publications in the United States (Figure S1). This interest, on the one hand, can be explained due to the advances made by fundamental research, in particular associated with deoxyribonucleic acid (DNA) sequencing, which improves our knowledge of the microbiota [20]. On the other hand, dysbiosis is becoming a threat to public health, and now is recognized as linked to several other chronic diseases such as inflammatory diseases as well as mental and neurological diseases [10, 11], highlighting its role in both gastrointestinal and mental health.

The legal status of the patent documents analyzed (Figure 1B) demonstrates the relative youth of innovation related to dysbiosis. In fact, 37% of patents are granted and are still active, and 50% of documents are still being vetted by the various IP offices.

According to the top 10 jurisdictions for the publication of processed patent documents (Figure 1C), publication in the United States is the most prolific with 4361 documents, accounting for almost 54% of all publications. Beyond the health issue of progress in the fight against dysbiosis, we can explain this observation by the importance of American investments in this field of research, supported by dozens of startups with funding of several 100 million dollars (www.medicalstartups.org/top/microbiome; accessed on December 17, 2024), and by the establishment of the most innovative companies in the field in the United States (Figure 1D). Indeed, seven of the top 10 companies as owners of dysbiosis patents have their headquarters in the United States. Psomagen Inc. is the leader in this field, with 171 patent documents. It provides genomics solutions, in particular clinical sequencing and research sequencing. Ubiome Inc. (San Francisco, CA, USA) is second with 155 patent documents. It is a provider of sequencing‐based clinical microbiome tests designed to collect and analyze data on the human microbiome. However, financial problems precipitated the transfer of its patent ownership and IP capital taken over by Psomagen Inc. since 2020. CJ Bioscience Inc. (Seoul, South Korea) is third in the owners' ranking with 146 patent documents. It is an Artificial Intelligence (AI)‐based microbiome drug development company interested in microbiome research at several levels, such as bioinformatics and the next‐generation sequencing.

Patent document data analysis reveals a set of diseases associated with dysbiosis‐centric innovation. The top five diseases (Figure 2A) are inflammatory diseases, infectious diseases, cancers, gastrointestinal diseases, and skin diseases [21, 22, 23, 24, 25, 26, 27, 28, 29, 30]. In the case of inflammatory diseases, a more precise analysis shows that 55% of the inflammation addressed by the patents derives from the bowel. This result is explained by the fact that dysbiosis leads to excessive activation of the immune system in the case of many gastrointestinal disorders, such as Crohn's disease or ulcerative colitis, which increases chronic inflammation. In the case of cancers, about 60% of patents claim innovations for the prevention and treatment of colorectal cancers. Indeed, the changes in the gut microbiota characteristics of dysbiosis increase the likelihood of developing colorectal cancer, via altering the intestinal barrier and inducing genetic mutations by certain products of pathogenic bacteria, such as *Escherichia coli* and *Fusobacterium nucleatum*. This discovery is in line with the bibliographical evidence and illustrates the advances of innovation in managing of the symptoms and effects of dysbiosis.

Regarding the 10 most cited patents (Figure 2B), we observe how preparations based on bacteria, or their derivatives act to mitigate the consequences of dysbiosis. Three out of the top 10 examined patents (US2014/0147425A1, US2014/0199281A1, and US9028841B2) belong to the same simple patent family. They describe therapeutic compositions containing purified populations of bacteria that, once administered to the target person, participate in preventing and treating the symptoms associated with dysbiosis. The most cited patents also concern compositions containing bacterial combinations beneficial for maintaining or restoring a healthy microbiota to regulate the immune process (WO2015/095241A2 and US2014/0341921A1). Other innovations show interest in manipulating genetic materials to develop diagnostic procedures or effective therapies (US8603749B2 and US2013/0121968A1).

The most cited scientific references in the patent documents (Figure 2C) express the importance of the precise and correct identification of microbial diversity and types of metabolism that come into play, especially in the different parts of the digestive system. These documents also describe some advances in the management of diseases related to microbial imbalance in the human body, including through the use of T‐cell lymphocytes, which is found in a patent application US2014/0341921A1, one of the most cited patent documents.

The examination of the IPC codes assigned to the patent documents analyzed confirms the observation from the analysis of the most cited patent documents, namely the action taken on bacteria and their derivatives to limit the effects of dysbiosis and cure it. Regarding the IPC codes, the code A61K35/74 is in the majority with 1483 citations. It refers to innovations in therapeutic preparations containing bacterial proteins, which shows the importance of bacteria and their derivatives in remedying dysbiosis. An examination of the Top 20 IPC codes allows us to identify the main areas of innovation claimed by the patents studied (Figure 2D). The first area is precisely that of the use of bacteria and their derivatives (with 3632 patent documents), which includes the use of probiotics (A61K35/741), spore‐forming bacteria (A61K35/742), lactic acid bacteria (A61K35/744), bifidobacteria (A61K35/745), and lactobacilli (A61K35/747). The second area, with 2231 patent documents, focuses on therapeutic activities and includes problems of the digestive system (A61P1/00), the development and use of antibacterial (A61P31/04), antineoplastic (A61P35/00), and antipyretic or anti‐inflammatory agents (A61P29/00). Other codes appear promising, such as C12N1/20 for the development of bacterial culture media, assigned to 779 documents; C12Q1/68 for nucleic acid measurements and tests and C12Q1/689 for nucleic acid products used for the detection or identification of bacteria, assigned to 351 and 461 documents, respectively; as well as codes A23L33/00 concerning the modification of the nutritional qualities of foods and A23L33/135 concerning the particular modification of foods with the use of bacteria, attributed to 306 and 703 documents, respectively.

Table S1 presents a selection of 10 patent documents. This selection represents the most relevant documents on the basis of a selection grid that classifies the documents collected from our exhaustive search, taking into account the relevance of the documents according to the method used by the source database of *The Lens*, as well as additional elements represented by the size of the simple and extended families of patents, their legal status, and their age.

These innovations are based on three major axes, which are therapies aimed at rebalancing the microbiota, the development and improvement of diagnostic tools, and the treatment of specific pathologies. Examples of innovations aimed at rebalancing the microbiota are most represented in our document selection. They mainly use approaches based on bacteria and prebiotics. The patent AU2018/204406B2 claims a synergistic bacterial composition capable of preventing and treating imbalances in the microbiota. Patent application US2017/0151269A1 describes the use of therapeutic glycans to modulate the gastrointestinal microbiota and treat dysbiosis. The EP3468573B1 patent presents a formulation of specific bacterial strains to combat *Clostridium difficile*. Patent application US2017/0281692A1 seeks to standardize microbiota restoration therapy from feces. Further, document number WO2020/048609A1 aims at the development of prebiotics enriched with a polysaccharide originating from some plants to restore intestinal balance.

Microbiota analysis and diagnostic technologies are also concerned by some of our selected documents. Advanced techniques allow for accurate and rapid identification of microbes, allowing for accurate diagnosis and personalized treatments. The European patent EP3265822B1 claims the invention to improve the rapid detection of microbial imbalances through the use of spectrometry to analyze the microbiome and its interactions with tissues. Patent application WO2018/053308A1 claims a universal method of microbial DNA extraction for optimizing the identification of pathogenic organisms and complex microbial populations. The U.S. patent US10347367B2 highlights the impact of the microbiome on cardiovascular function to plan personalized treatments. Other innovations harness microbiota to treat specific conditions, as illustrated in our selection by patent application US2024/0066079A1, which claims a natural alternative (*Propionibacterium acnes*) to conventional acne treatments. Further, the patent application EP3616678A1 claims the development of a topical composition based on glycerin and *Pichia anomala* extract to improve skin microflora. These advances contribute to a better understanding of the link between the microbiome and health and also pave the way for personalized and more precise therapeutic approaches.

## CLINICAL TRIAL OUTCOMES AND REAL‐WORLD EVIDENCE

A critical aspect of validating microbiome‐based therapies for intestinal dysbiosis involves synthesizing clinical trial outcomes and real‐world evidence. Current therapeutic strategies, such as probiotics, fecal microbiota transplantation (FMT), and microbiome‐targeted drugs, have shown varying degrees of efficacy in restoring microbial balance and alleviating disease symptoms. Clinical trial registries, such as ClinicalTrials.gov (https://clinicaltrials.gov/search?cond=Dysbiosis; accessed on March 8, 2025), provides extensive data on ongoing and completed trials that evaluate the efficacy of these treatments.

Probiotics have demonstrated potential in modulating gut microbiota, particularly in reducing inflammation and improving gut barrier function in inflammatory bowel diseases (IBD). For example, clinical trials have consistently reported that probiotic strains such as *Lactobacillus* and *Bifidobacterium* show promising outcomes in reducing symptoms of ulcerative colitis and Crohn's disease. Similarly, FMT has gained attraction as a treatment for *Clostridioides difficile* infections, and ongoing trials are exploring its application in broader dysbiosis‐related disorders, including metabolic syndromes and inflammatory diseases. Along this line, searching PubMed revealed up to date (as of March 9, 2025) over a 500 published meta‐analysis of clinical trial studies exploring the effectiveness of probiotics in different health conditions, and readers are referred to this literature pool for more detailed insights.

## FUTURE RESEARCH DIRECTIONS AND THERAPEUTIC HORIZONS IN DYSBIOSIS

### Advancing gene editing for microbiota modulation

The advent of clustered regularly interspaced short palindromic repeats (CRISPR)‐Cas9 and other gene‐editing technologies has revolutionized microbiome research, providing precise tools to manipulate gut microbial populations for therapeutic applications. Future research should focus on leveraging CRISPR‐based editing to engineer commensal bacteria capable of mitigating dysbiosis‐associated diseases. Targeted genetic modifications can enable probiotic strains to enhance beneficial metabolite production, degrade pathogenic compounds, or promote restoration of microbial balance. For example, engineered *Bacteroides* strains could be programmed to produce SCFAs that enhance gut barrier integrity and modulate immune responses, thereby mitigating IBD and metabolic disorders.

### Integrating T‐cell lymphocyte‐based therapeutics

A particularly exciting relevant development is the integration of T‐cell lymphocyte‐based therapeutics with synthetic microbiome therapies. T‐cell engineering, like the chimeric antigen receptor (CAR)‐T cell technology, has demonstrated remarkable success in immunotherapy, and its application to microbiome‐associated disorders is gaining increasing interest. Engineered T‐cells can be designed to recognize and respond to gut dysbiosis‐related fluctuations of relevant biomolecules by modulating immune responses in real time. For example, synthetic biology approaches can enable T‐cells to detect inflammatory signals associated with dysbiosis and subsequently release targeted cytokines to restore gut homeostasis. Conversely, recent research has indicated that microbiota composition is able to influence the success or toxicity of the CAR‐T therapy, opening the possibility for simultaneous targeting microbiota to achieve superior CAR‐T therapy outcomes. Thus, by combining immune‐based interventions with precision microbiome‐targeting approaches, researchers can develop more comprehensive and adaptive therapeutic strategies for diverse conditions such as IBD and autoimmune disorders.

### Overcoming challenges and ethical considerations

The rapid commercialization of microbiome‐based therapies presents significant ethical and regulatory challenges, particularly regarding equitable access, cost‐related disparities, and patent monopolies. While these therapies hold great promise, their high development costs and limited insurance coverage create affordability barriers, disproportionately affecting low‐income populations. Patent monopolization by biotech firms further restricts competition and drives up costs, highlighting the need for open‐access research initiatives, patent‐sharing models, and non‐profit‐driven innovations to ensure broader availability. Regulatory inconsistencies across jurisdictions also pose hurdles, with microbiome therapies classified variably as biological drugs, supplements, or novel treatments, leading to delays in approval and potential safety risks. The lack of standardized global guidelines underscores the urgency of harmonizing regulatory frameworks, ensuring rigorous safety assessments, and establishing ethical guidelines for engineered microbiota and CRISPR‐based interventions. Addressing these concerns requires collaborative efforts from policymakers, researchers, and industry leaders to develop pricing strategies, public health programs, and accessible microbiome innovations that prioritize patient well‐being over commercial interests. By fostering balanced policies and equitable deployment, microbiome‐based therapies can evolve into transformative yet responsibly managed healthcare solutions.

## CONCLUSION

In this study, qualitative and quantitative data from patent documents on dysbiosis were processed to perform a comprehensive analysis that can be useful in understanding the technological innovation developed over the last 20 years, aimed at reducing the symptoms of dysbiosis and related diseases. Although the technical area addressed is a young field of innovation, with most patents still in force or under examination, the growing trend of patent filings related to dysbiosis is confirmed. The United States is becoming a leader in the claim of patented processes and methods, drawn by companies and investment funds with solid finances. Dysbiosis is associated with several other diseases; sometimes, it is their cause, and sometimes, it appears to be their consequence. The remediation of the microbial imbalance observed during dysbiosis requires the perfect identification of the microbial and particularly bacterial species involved, which has been made possible thanks to new genetic manipulation techniques. In addition, the most cited patents reveal the importance of using purified bacterial strains to manage dysbiosis and its effects. The knowledge gained through the present analysis might help accelerate the development of effective solutions, inform regulatory and market strategies, and help translate microbiome science into impactful health interventions. The convergence of gene editing, synthetic biology, and microbiome research holds immense promises for revolutionizing the treatment of dysbiosis and its associated pathologies. Future research should prioritize the development of precision microbiome therapeutics, noninvasive diagnostic tools, and robust regulatory frameworks to facilitate the safe translation of these innovations into clinical practice. By harnessing these cutting‐edge technologies, the field can move closer to personalized and highly targeted microbiota‐based precision interventions for a range of gastrointestinal and systemic diseases.

## AUTHOR CONTRIBUTIONS

**Reda El Boukhari**: Conceptualization; methodology; investigation; writing—original draft; validation; formal analysis; visualization. **Maima Matin**: Writing—review and editing; formal analysis; validation. **Latifa Bouissane**: Writing—review and editing; validation; formal analysis. **Michał Ławiński**: Validation; writing—review and editing; formal analysis. **Oleh Lushchak**: Validation; writing—review and editing; formal analysis. **Rajeev K. Singla**: Validation; writing—review and editing; formal analysis. **Michel‐Edwar Mickael**: Formal analysis; validation; writing—review and editing. **Jordi Mayneris‐Perxachs**: Validation; writing—review and editing; formal analysis. **Maria Eleni Grafakou**: Formal analysis; Validation; Writing—review and editing. **Shuhua Xu**: Formal analysis; validation; writing—review and editing. **Bowen Liu**: Validation; writing—review and editing; formal analysis. **Jiayi Guan**: Validation; formal analysis; writing—review and editing. **Andrzej Półtorak**: Validation; writing—review and editing; formal analysis. **Arkadiusz Szpicer**: Validation; writing—review and editing; formal analysis. **Agnieszka Wierzbicka**: Validation; writing—review and editing; formal analysis. **Nikolay T. Tzvetkov**: Formal analysis; validation; writing—review and editing. **Maciej Banach**: Formal analysis; writing—review and editing; validation. **Jarosław Olav Horbańczuk**: Formal analysis; Validation; writing—review and editing. **Artur Jóźwik**: Formal analysis; writing—review and editing; validation. **Marco Cascella**: Validation; writing—review and editing; formal analysis. **Bairong Shen**: Formal analysis; writing—review and editing; validation. **Vasil Radoslavov Pirgozliev**: Validation; writing—review and editing; formal analysis. **Dongdong Wang**: Formal analysis; validation; writing—review and editing. **Olena Litvinova**: Validation; formal analysis; writing—review and editing. **Olga Adamska**: Writing—review and editing; validation; formal analysis. **Agnieszka Kamińska**: Validation; writing—review and editing; formal analysis. **Marcin Łapiński**: Formal analysis; validation; writing—review and editing. **Artur Stolarczyk**: Formal analysis; writing—review and editing; validation. **Ioana Berindan‐Neagoe**: Formal analysis; writing—review and editing; validation. **Luigi Milella**: Formal analysis; writing—review and editing; validation. **Andy Wai Kan Yeung**: Formal analysis; validation; writing—review and editing. **Prashanth Suravajhala**: Validation; writing—review and editing; formal analysis. **Anupam Bishayee**: Validation; writing—review and editing; formal analysis. **Ronan Lordan**: Writing—review and editing; validation; formal analysis. **Laszlo Barna Iantovics**: Formal analysis; writing—review and editing; validation. **Ricardo Lagoa**: Formal analysis; writing—review and editing; validation. **Monika Michalczuk**: Formal analysis; writing—review and editing; validation. **Jivko Stoyanov**: Formal analysis; writing—review and editing; validation. **A Douglas Kinghorn**: Formal analysis; validation; writing—review and editing. **Banaz Jalil**: Validation; formal analysis; writing—review and editing. **Wolfram Weckwerth**: Formal analysis; writing—review and editing; validation. **Bey Hing Goh**: Formal analysis; writing—review and editing; validation. **Meng‐Yao Li**: Formal analysis; writing—review and editing; validation. **Gyaneshwer Chaubey**: Writing—review and editing; formal analysis; validation. **Gian Luigi Russo**: Formal analysis; writing—review and editing; validation. **Sara Frazzini**: Formal analysis; writing—review and editing; validation. **Luciana Rossi**: Formal analysis; writing—review and editing; validation. **Maurizio Battino**: Formal analysis; validation; writing—review and editing. **Wei Jia**: Validation; writing—review and editing; formal analysis. **Qi Su**: Validation; writing—review and editing; formal analysis. **Xiaoqiang Ma**: Writing—review and editing; validation; formal analysis. **Judith M. Rollinger**: Validation; writing—review and editing; formal analysis. **Simon K.‐M. R. Rittmann**: Validation; formal analysis; writing—review and editing. **Helen Sheridan**: Formal analysis; writing—review and editing; validation. **John J Walsh**: Formal analysis; writing—review and editing; validation. **Gérard Lizard**: Formal analysis; writing—review and editing; validation. **Tomasz M. Karpiński**: Formal analysis; writing—review and editing; validation. **Ana Sanches Silva**: Validation; writing—review and editing; formal analysis. **Jakub Piwowarski**: Validation; writing—review and editing; formal analysis. **Liwei Xie**: Validation; writing—review and editing; formal analysis. **Tai‐Ping Fan**: Validation; writing—review and editing; formal analysis. **Francesca Giampieri**: Validation; formal analysis; writing—review and editing. **Adil El Midaoui**: Writing—review and editing; validation; formal analysis. **Ka‐Hing Wong**: Writing—review and editing; validation; formal analysis. **Ren‐You Gan**: Project administration; validation; writing—review and editing; conceptualization; methodology; formal analysis; supervision; resources. **Ahmed Fatimi**: Data curation; supervision; resources; project administration; formal analysis; validation; writing—review and editing; conceptualization; methodology. **Atanas G. Atanasov**: Resources; supervision; project administration; writing—review and editing; validation; conceptualization; methodology; formal analysis.

## CONFLICT OF INTEREST STATEMENT

The authors declare no conflicts of interest. The authors have no relevant affiliations or financial involvement with any organization or entity with a financial interest in or financial conflict with the subject matter or materials discussed in this article.

## ETHICS STATEMENT

No animals or humans were involved in this study.

## Supporting information


**Figure S1:** Dysbiosis patent documents publication over time in the three leading jurisdictions. The trends highlight the growing interest in dysbiosis‐related innovations, particularly in the United States, which has consistently led in patent filings. The recent decline observed in 2023‐2024 may indicate market saturation, shifts in research focus, or delays in patent processing. WIPO, World Intellectual Property Organization.

**Table S1:** Selection of 10 relevant dysbiosis patent documents.

## Data Availability

The data that support the findings of this study are openly available in Data set of patents related to dysbiosis at https://doi.org/10.5281/zenodo.14984713, reference number 14984713. The data presented in this study are available within this article's content. To enhance raw data accessibility, a tabulation of the surveyed patents—as supporting information—can be downloaded at: https://doi.org/10.5281/zenodo.14984713. Supplementary materials (figures, tables, graphical abstract, slides, videos, Chinese translated version, and updated materials) may be found in the online DOI or iMeta Science http://www.imeta.science/.

## References

[1] Parkin, Kimberley , Claus T. Christophersen , Valerie Verhasselt , Matthew N. Cooper , and David Martino . 2021. “Risk Factors for Gut Dysbiosis in Early Life.” Microorganisms 9(10): 2066. 10.3390/microorganisms9102066 34683389 PMC8541535

[2] Sharma, Kunal , and Kunal Kumar . 2023. “Dysbiosis and Its Varied Impacts.” Eds. N. Srivastava , S. Ibrahim , J. Chattopadhyay , and M. Arbab . in The Gut Microbiota in Health and Disease, Hoboken, NJ: Wiley, pp. 41–54. 10.1002/9781119904786.ch5

[3] Hutyra, Ferenc , Josef Marek , and Rezsö Manninger . 1949. Special Pathology and Therapeutics of the Diseases of Domestic Animals. Bailliere, Tindall and Cox Madison, WI. https://books.google.com/books?id=afnhAAAAMAAJ

[4] Hooks, Katarzyna B. , and Maureen A. O'Malley . 2017. “Dysbiosis and Its Discontents.” mBio 8(5): 17. 10.1128/mbio.01492-17 PMC5635691 29018121

[5] Tomasello, Giovanni , Margherita Mazzola , Angelo Leone , Emanuele Sinagra , Giovanni Zummo , Felicia Farina , Provvidenza Damiani , et al. 2016. “Nutrition, Oxidative Stress and Intestinal Dysbiosis: Influence of Diet on Gut Microbiota in Inflammatory Bowel Diseases.” Biomedical Papers 160(4): 461–466. 10.5507/bp.2016.052 27812084

[6] Carías Domínguez, Ailim Margarita , Dimas de Jesús Rosa Salazar , Juan Pablo Stefanolo , Maria Claudia Cruz Serrano , Isabel Cristina Casas , and Julio Ricardo Zuluaga Peña . 2024. “Intestinal Dysbiosis: Exploring Definition, Associated Symptoms, and Perspectives for a Comprehensive Understanding—a Scoping Review.” Probiotics and Antimicrobial Proteins 17(1): 440–449. 10.1007/s12602-024-10353-w 39235661 PMC11832579

[7] Gagliardi, Antonella , Valentina Totino , Fatima Cacciotti , Valerio Iebba , Bruna Neroni , Giulia Bonfiglio , Maria Trancassini , Claudio Passariello , Fabrizio Pantanella , and Serena Schippa . 2018. “Rebuilding the Gut Microbiota Ecosystem.” International Journal of Environmental Research and Public Health 15(8): 1679. 10.3390/ijerph15081679 30087270 PMC6121872

[8] Thomas, Andrew Maltez , Marine Fidelle , Bertrand Routy , Guido Kroemer , Jennifer A. Wargo , Nicola Segata , and Laurence Zitvogel . 2023. “Gut OncoMicrobiome Signatures (GOMS) as Next‐Generation Biomarkers for Cancer Immunotherapy.” Nature Reviews Clinical Oncology 20(9): 583–603. 10.1038/s41571-023-00785-8 PMC11258874 37365438

[9] Dréno, Brigitte , Marie Ange Dagnelie , Amir Khammari , and Stéphane Corvec . 2020. “The Skin Microbiome: A New Actor in Inflammatory Acne.” American Journal of Clinical Dermatology 21(Suppl 1): 18–24. 10.1007/s40257-020-00531-1 32910436 PMC7584556

[10] Xiong, Ruo‐Gu , Jiahui Li , Jin Cheng , Dan‐Dan Zhou , Si‐Xia Wu , Si‐Yu Huang , Adila Saimaiti , Zhi‐Jun Yang , Ren‐You Gan , and Hua‐Bin Li . 2023. “The Role of Gut Microbiota in Anxiety, Depression, and Other Mental Disorders as Well as the Protective Effects of Dietary Components.” Nutrients 15(14), 3258. 10.3390/nu15143258 37513676 PMC10384867

[11] Korf, Janelle M. , Bhanu P. Ganesh , and Louise D. McCullough . 2022. “Gut Dysbiosis and Age‐Related Neurological Diseases in Females.” Neurobiology of Disease 168(n/a), 105695. 10.1016/j.nbd.2022.105695 35307514 PMC9631958

[12] Hou, Kaijian , Zhuo‐Xun Wu , Xuan‐Yu Chen , Jing‐Quan Wang , Dongya Zhang , Chuanxing Xiao , Dan Zhu , et al. 2022. “Microbiota in Health and Diseases.” Signal Transduction and Targeted Therapy 7(1): 135. 10.1038/s41392-022-00974-4 35461318 PMC9034083

[13] Winter, Sebastian E. , and Andreas J. Bäumler . 2023. “Gut Dysbiosis: Ecological Causes and Causative Effects on Human Disease.” Proceedings of the National Academy of Sciences of the United States of America 120(50), e2316579120. 10.1073/pnas.2316579120 38048456 PMC10722970

[14] Vamanu, Emanuel , and Sachchida Nand Rai . 2021. “The Link between Obesity, Microbiota Dysbiosis, and Neurodegenerative Pathogenesis.” Diseases 9(3): 45. 10.3390/diseases9030045 34201465 PMC8293145

[15] Llenos, Luzz Claire P. , Ivy Lorelei G. Miranda , Andrei A. Domogo , and Juancho A. Collera . 2023. “Mathematical Modeling of Antibiotic Resistance in Hospital With Dysbiosis.” Chiang Mai Journal of Science 50(3), e2023027. 10.12982/cmjs.2023.027

[16] Liu, Xiangyi , Haiyi Zhao , and Aloysius Wong . 2024. “Accounting for the Health Risk of Probiotics.” Heliyon 10(6), e27908. 10.1016/j.heliyon.2024.e27908 38510031 PMC10950733

[17] Aagaard, Kjersti , and Elizabeth Hohmann . 2019. “Regulating Microbiome Manipulation.” Nature Medicine 25(6): 874–876. 10.1038/s41591-019-0451-1 31133692

[18] Merenstein, Daniel , Bruno Pot , Gregory Leyer , Arthur C. Ouwehand , Geoffrey A. Preidis , Christopher A. Elkins , Colin Hill , et al. 2023. “Emerging Issues in Probiotic Safety: 2023 Perspectives.” Gut Microbes 15(1), 2185034. 10.1080/19490976.2023.2185034 36919522 PMC10026873

[19] Matin, Maima , Dalibor Hrg , Olena Litvinova , Małgorzata Łysek‐Gładysinska , Agnieszka Wierzbicka , Jarosław Olav Horbańczuk , Artur Jóźwik , and Atanas G. Atanasov . 2024. “The Global Patent Landscape of Functional Food Innovation.” Nature Biotechnology 42(10): 1493–1497. 10.1038/s41587-024-02410-0 39402344

[20] Behjati, Sam , and Patrick S. Tarpey . 2013. “What Is Next Generation Sequencing?” Archives of Disease in Childhood‐Education & Practice Edition 98(6): 236–238. 10.1136/archdischild-2013-304340 23986538 PMC3841808

[21] Frank, Daniel N. , Allison L. Amand, St. , Robert A. Feldman , Edgar C. Boedeker , Noam Harpaz , and Norman R. Pace . 2007. “Molecular‐Phylogenetic Characterization of Microbial Community Imbalances in Human Inflammatory Bowel Diseases.” Proceedings of the National Academy of Sciences of the United States of America 104(34): 13780–13785. 10.1073/pnas.0706625104 17699621 PMC1959459

[22] Sokol, Harry , Bénédicte Pigneur , Laurie Watterlot , Omar Lakhdari , Luis G. Bermúdez‐Humarán , Jean‐Jacques Gratadoux , Sébastien Blugeon , et al. 2008. “ *Faecalibacterium prausnitzii* is an Anti‐inflammatory Commensal Bacterium Identified by Gut Microbiota Analysis of Crohn Disease Patients.” Proceedings of the National Academy of Sciences of the United States of America 105(43): 16731–16736. 10.1073/pnas.0804812105 18936492 PMC2575488

[23] Atarashi, Koji , Takeshi Tanoue , Kenshiro Oshima , Wataru Suda , Yuji Nagano , Hiroyoshi Nishikawa , Shinji Fukuda , et al. 2013. “T_reg_ Induction by a Rationally Selected Mixture of *Clostridia* Strains From the Human Microbiota.” Nature 500(7461): 232–236. 10.1038/nature12331 23842501

[24] Eckburg, Paul B. , Elisabeth M. Bik , Charles N. Bernstein , Elizabeth Purdom , Les Dethlefsen , Michael Sargent , Steven R. Gill , Karen E. Nelson , and David A. Relman . 2005. “Diversity of the Human Intestinal Microbial Flora.” Science 308(5728): 1635–1638. 10.1126/science.1110591 15831718 PMC1395357

[25] Atarashi, Koji , Takeshi Tanoue , Tatsuichiro Shima , Akemi Imaoka , Tomomi Kuwahara , Yoshika Momose , Genhong Cheng , et al. 2011. “Induction of Colonic Regulatory T Cells by Indigenous *Clostridium* Species.” Science 331(6015): 337–341. 10.1126/science.1198469 21205640 PMC3969237

[26] Altschul, Stephen F. , Warren Gish , Webb Miller , Eugene W. Myers , and David J. Lipman . 1990. “Basic Local Alignment Search Tool.” Journal of Molecular Biology 215(3): 403–410. 10.1016/S0022-2836(05)80360-2 2231712

[27] Sokol, H. , P. Seksik , J. P. Furet , O. Firmesse , I. Nion‐Larmurier , L. Beaugerie , J. Cosnes , G. Corthier , P. Marteau and J. Doré . 2009. “Low Counts of *Faecalibacterium prausnitzii* in Colitis Microbiota.” Inflammatory Bowel Diseases 15(8): 1183–1189. 10.1002/ibd.20903 19235886

[28] Louis, Petra , and Harry J. Flint . 2009. “Diversity, Metabolism and Microbial Ecology of Butyrate‐Producing Bacteria From the Human Large Intestine.” FEMS Microbiology Letters 294(1): 1–8. 10.1111/j.1574-6968.2009.01514.x 19222573

[29] Gaboriau‐Routhiau, Valérie , Sabine Rakotobe , Emelyne Lécuyer , Imke Mulder , Annaïg Lan , Chantal Bridonneau , Violaine Rochet , et al. 2009. “The Key Role of Segmented Filamentous Bacteria in the Coordinated Maturation of Gut Helper T Cell Responses.” Immunity 31(4): 677–689. 10.1016/j.immuni.2009.08.020 19833089

[30] Huttenhower, Curtis , Dirk Gevers , Rob Knight , Sahar Abubucker , Jonathan H. Badger , Asif T. Chinwalla , Heather H. Creasy , et al. 2012. “Structure, Function and Diversity of the Healthy Human Microbiome.” Nature 486(7402): 207–214. 10.1038/nature11234 22699609 PMC3564958

