# Supplementary material for: Enhancing human gut health: Global innovations in dysbiosis management
Source: Imeta. 2025 Apr 13;4(3):e70028. doi: 10.1002/imt2.70028 (PMC12130569; doi:10.1002/imt2.70028)
Supplement: Supplementary file 1 — Figure S1: Dysbiosis patent documents publication over time in the three leading jurisdictions. The trends highlight the growing interest in dysbiosis‐related innovations, particularly in the United States, which has consistently led in patent filings. The recent decline observed in 2023‐2024 may indicate market saturation, shifts in research focus, or delays in patent processing. WIPO, World Intellectual Property Organization. [file IMT2-4-e70028-s002.docx]

Supporting information

**Enhancing human gut health: global innovations in dysbiosis management**

**Running title: The global patent landscape of innovations for counteracting dysbiosis**

Reda El Boukhari^1^, Maima Matin^2^, Latifa Bouissane^3^, Michał Ławiński^2,4^, Oleh Lushchak^5,6^, Rajeev K. Singla^7,8^, Michel-Edwar Mickael^2^, Jordi Mayneris-Perxachs^9,10,11^, Maria Eleni Grafakou^12^, Shuhua Xu^13,14^, Bowen Liu^15^, Jiayi Guan^16^, Andrzej Półtorak^17^, Arkadiusz Szpicer^17^, Agnieszka Wierzbicka^2,17^, Nikolay T. Tzvetkov^18^, Maciej Banach^19,20,21,22^, Jarosław Olav Horbańczuk^2^, Artur Jóźwik^2^, Marco Cascella^23^, Bairong Shen^24,25^, Vasil Radoslavov Pirgozliev^26^, Dongdong Wang^27,28^, Olena Litvinova^29,30^, Olga Adamska^31^, Agnieszka Kamińska^31^, Marcin Łapiński^32^, Artur Stolarczyk^32^, Ioana Berindan-Neagoe^33^, Luigi Milella^34^, Andy Wai Kan Yeung^35^, Prashanth Suravajhala^36,37^, Anupam Bishayee^38^, Ronan Lordan^39^, Laszlo Barna Iantovics^40^, Ricardo Lagoa^41,42^, Monika Michalczuk^43^, Jivko Stoyanov^44,45^, A. Douglas Kinghorn^46^, Banaz Jalil^47^, Wolfram Weckwerth^48,49^, Bey Hing Goh^50,51,52^, Meng-Yao Li^53,54^, Gyaneshwer Chaubey^55^, Gian Luigi Russo^56^, Sara Frazzini^57^, Luciana Rossi^57^, Maurizio Battino^58,59,60^, Wei Jia^61^, Qi Su^62,63^, Xiaoqiang Ma^64^, Judith M. Rollinger^65^, Simon K.-M. R. Rittmann^66^, Helen Sheridan^67,68^, John J. Walsh^67^, Gérard Lizard^68^, Tomasz M. Karpiński^69^, Ana Sanches Silva^70,71^, Jakub Piwowarski^72^, Liwei Xie^73,74^, Tai-Ping Fan^74^, Francesca Giampieri^58,59,75,76^, Adil El Midaoui^77,78^, Ka-Hing Wong^79,80^, Ren-You Gan^79,80*^, Ahmed Fatimi^1*^, Atanas G. Atanasov^2,30,81*^

*^1^ Chemical Science and Engineering Research Team (ERSIC), Department of Chemistry, Polydisciplinary Faculty of Beni Mellal (FPBM), Sultan Moulay Slimane University (USMS), P.O. Box 592 Mghila, Beni Mellal 23000, Morocco*

*^2^ Institute of Genetics and Animal Biotechnology of the Polish Academy of Sciences, Jastrzebiec, 05-552 Magdalenka, Poland*

*^3^ Laboratory of Molecular Chemistry, Materials and Catalysis, Faculty of Sciences and Techniques, Sultan Moulay Slimane University, Beni Mellal 23000, Morocco*

*^4^ Department of General, Gastroenterologic and Oncologic Surgery, Medical University of Warsaw, 02-097 Warsaw, Poland*

*^5^ Department of Biochemistry and Biotechnology, Vasyl Stefanyk Precarpathian National University, Ivano-Frankivsk 76000, Ukraine*

*^6^ Research and Development University, Ivano-Frankivsk 76000, Ukraine*

*^7^ Department of Pharmacy and Institutes for Systems Genetics, Center for High Altitude Medicine, Frontiers Science Center for Disease-related Molecular Network, West China Hospital, Sichuan University, Chengdu, Sichuan 610041, China*

*^8^ School of Pharmaceutical Sciences, Lovely Professional University, Phagwara, Punjab 144411, India*

*^9^ Department of Diabetes, Endocrinology and Nutrition, Dr. Josep Trueta University Hospital, Girona 17007, Spain*

*^10^ CIBER Fisiopatología de la Obesidad y Nutrición (CIBERobn), Madrid 28029, Spain*

*^11^ Integrative Systems Medicine and Biology Group, Girona Biomedical Research Institute (IDIBGI-CERCA), Parc Hospitalari Martí i Julià, Edifici M2, Salt 17190, Spain*

*^12^ Chair of Pharmaceutical Biology, Faculty of Pharmacy and Chemistry, University of Regensburg 93053, Germany*

*^13^ Center for Evolutionary Biology, School of Life Sciences, Fudan University, Shanghai 200438, China*

*^14^ Human Phenome Institute, Zhangjiang Fudan International Innovation Center, Fudan University, Shanghai 201210, China*

*^15^ School of Agriculture, Yunnan University, Kunming 650204, China*

*^16^ Henan Institute of Medical and Pharmaceutical Sciences, Zhengzhou University, Zhengzhou 450001, China*

*^17^ Department of Technique and Food Development, Institute of Human Nutrition Sciences, Warsaw University of Life Sciences, Nowoursynowska Str. 159 C, 02-776 Warsaw, Poland*

*^18^ Department of Biochemical Pharmacology and Drug Design, Institute of Molecular Biology “Roumen Tsanev”, Bulgarian Academy of Sciences, Sofia 1113, Bulgaria*

*^19^ Faculty of Medicine, The John Paul II Catholic University of Lublin (KUL), 20-950 Lublin, Poland*

*^20^ Department of Cardiology and Adult Congenital Heart Diseases, Polish Mother’s Memorial Hospital Research Institute (PMMHRI), 93-338 Lodz, Poland*

*^21^ Department of Preventive Cardiology and Lipidology, Medical University of Lodz (MUL), 93-338 Lodz, Poland*

*^22^ Ciccarone Center for the Prevention of Cardiovascular Disease, Johns Hopkins University School of Medicine, Baltimore, MD, 21205, USA*

*^23^ Anesthesia and Pain Medicine, Department of Medicine, Surgery and Dentistry "Scuola MedicaSalernitana", University of Salerno, Baronissi 84081, Italy*

*^24^ Joint Laboratory of Artificial Intelligence for Critical Care Medicine, Department of Critical Care Medicine and Institutes for Systems Genetics, Frontiers Science Center for Disease-related Molecular Network, West China Hospital, Sichuan University, Chengdu, Sichuan 610093, China*

*^25^ Center for High Altitude Medicine, West China Hospital, Sichuan University, Chengdu, Sichuan 610041, China*

*^26^ National Institute of Poultry Husbandry, Harper Adams University, Newport TF10 8NB, United Kingdom*

*^27^ Centre for Metabolism, Obesity and Diabetes Research, McMaster University, Hamilton, ON, L8S 4K1, Canada*

*^28^ Division of Endocrinology and Metabolism, Department of Medicine, McMaster University, Hamilton, ON, L8S 4K1, Canada*

*^29^ National University of Pharmacy of the Ministry of Health of Ukraine, Kharkiv 61002, Ukraine*

*^30^ Ludwig Boltzmann Institute Digital Health and Patient Safety, Medical University of Vienna, Vienna 1180, Austria*

*^31^ Faculty of Medicine, Collegium Medicum Cardinal Stefan Wyszyński University in Warsaw, 01-938 Warsaw, Poland*

*^32^ Orthopaedic and Rehabilitation Department, Medical University of Warsaw, 01-938 Warsaw, Poland*

*^33^ Doctoral School Iuliu Hatieganu University of Medicine and Pharmacy, Cluj-Napoca, Romania Academy of Medical Sciences, Cluj-Napoca 400347, Romania*

*^34^ Department of Health Sciences, University of Basilicata, Via dell’Ateneo Lucano 10, Potenza 85100, Italy*

*^35^ Oral and Maxillofacial Radiology, Applied Oral Sciences and Community Dental Care, Faculty of Dentistry. The University of Hong Kong, Pokfulam, Hong Kong SAR*

*^36^ Amrita School of Biotechnology, Amrita Viswa Vidyapeetham, Clappana, Kerala 690525, India*

*^37^ Bioclues.org, Hyderabad 501511, India*

*^38^ Department of Pharmacology, College of Osteopathic Medicine, Lake Erie College of Osteopathic Medicine, Bradenton, FL, 34211, USA*

*^39^ The Institute for Translational Medicine and Therapeutics, Perelman School of Medicine, University of Pennsylvania, Philadelphia, PA, 19104, USA*

*^40^ Department of Electrical Engineering and Information Technology, George Emil Palade University of Medicine, Pharmacy, Science, and Technology of Targu Mures, Targu Mures, 540139, Romania*

*^41^ ESTG-Polytechnic Institute of Leiria, Morro do Lena-Alto do Vieiro, 2411-901 Leiria, Portugal*

*^42^ LSRE-LCM-Associate Laboratory in Chemical Engineering, University of Porto, Rua Dr. Roberto Frias, 4200-465 Porto, Portugal*

*^43^ Department of Animal Breeding, Institute of Animal Sciences, Warsaw University of Life Sciences - SGGW, Ciszewskiego 8 St., 02-786 Warsaw, Poland*

*^44^ Swiss Paraplegic Research, 6207 Nottwil, Switzerland*

*^45^ Institute of Social and Preventive Medicine (ISPM), University of Bern, 3012 Bern, Switzerland*

*^46^ College of Pharmacy, Ohio State University, Columbus, OH 43210, USA*

*^47^ Pharmacognosy and Phytotherapy, UCL School of Pharmacy, London WC1N 1AX, United Kingdom*

*^48^ Molecular Systems Biology Lab (MOSYS), Department of Functional and Evolutionary Ecology, University of Vienna, Vienna 1030, Austria*

*^49^ Vienna Metabolomics Center (VIME), University of Vienna, Vienna 1030, Austria*

*^50^ Sunway Biofunctional Molecules Discovery Centre (SBMDC), School of Medical and Life Sciences, Subang Jaya 47100, Malaysia*

*^51^ Biofunctional Molecule Exploratory (BMEX) Research Group, School of Pharmacy, Monash University Malaysia, Subang Jaya 47100, Malaysia*

*^52^ Faculty of Health, Australian Research Centre in Complementary and Integrative Medicine, University of Technology Sydney, Ultimo, NSW 2007, Australia*

*^53^ State Key Laboratory of Systems Medicine for Cancer, Shanghai Cancer Institute, Renji Hospital, Shanghai Jiao Tong University School of Medicine, Shanghai 200127, China*

*^54^ Department of Biliary-Pancreatic Surgery, Renji Hospital, Shanghai Jiao Tong University School of Medicine, Shanghai 200127, China*

*^55^ Cytogenetics Laboratory, Department of Zoology, Banaras Hindu University, Varanasi, Uttar Pradesh 221005, India*

*^56^ National Research Council, Institute of Food Sciences, Via Roma, 64, Avellino 83100, Italy*

*^57^ Department of Veterinary Medicine and Animal Science (DIVAS), University of Milan, Via dell’Università 6, Lodi 26900, Italy*

*^58^ Department of Clinical Sciences, Polytechnic University of Marche, Via Pietro Ranieri 65, Ancona 60131, Italy*

*^59^ Joint Laboratory on Food Science, Nutrition, and Intelligent Processing of Foods, Polytechnic University of Marche (Italy), Universidad Europea del Atlántico (Spain), and Jiangsu University (China), Ancona 60131, Italy.*

*^60^ International Joint Research Laboratory of Intelligent Agriculture and Agri-Products Processing, Jiangsu University, Zhenjiang 212013, China*

*^61^ Department of Pharmacology and Pharmacy, The University of Hong Kong, Pokfulam, Hong Kong SAR*

*^62^ Microbiota I-Center, 17 Science Park West Avenue, Hong Kong Science Park, Shatin, Hong Kong SAR*

*^63^ Department of Medicine and Therapeutics. The Chinese University of Hong Kong, Shatin, Hong Kong SAR*

*^64^ Department of Food Science and Technology, School of Agriculture and Biology, Shanghai Jiao Tong University, Shanghai 200240, China*

*^65^ Division of Pharmacognosy, Department of Pharmaceutical Sciences, Faculty of Life Sciences, University of Vienna, Vienna 1090, Austria*

*^66^ Archaea Physiology & Biotechnology Group, Department of Functional and Evolutionary Ecology, University of Vienna, Vienna 1030, Austria*

*^67^ The NatPro Centre & School of Pharmacy and Pharmaceutical Sciences. Trinity College Dublin, D02 PN40 Dublin, Ireland*

*^68^ PHYNOHA Consulting, 8, route de Daix, 21121 Fontaine-lès-Dijon, France*

*^69^ Department of Medical Microbiology, Poznań University of Medical Sciences, 60-512 Poznań, Poland*

*^70^ University of Coimbra, Faculty of Pharmacy, Polo III, Azinhaga de Santa Comba, 3000-548 Coimbra, Portugal*

*^71^ Centre for Animal Science Studies (CECA), ICETA, University of Porto, 4501-401 Porto, Portugal*

*^72^ Microbiota Lab, Department of Pharmaceutical Microbiology and Bioanalysis, Medical University of Warsaw, 02-091 Warsaw, Poland*

*^73^ State Key Laboratory of Applied Microbiology Southern China, Guangdong Provincial Key Laboratory of Microbial Culture Collection and Application, Guangdong Open Laboratory of Applied Microbiology, Institute of Microbiology, Guangdong Academy of Sciences, Guangzhou 510070, China*

*^74^ School of Life & Health Sciences, Fuyao University of Science & Technology, Fuzhou, Fujian 350001, China*

*^75^ Research Group on Food, Nutritional Biochemistry and Health, Universidad Europea del Atlántico, Isabel Torres 21, Santander 39011, Spain*

*^76^ International Research Center for Food Nutrition and Safety, Jiangsu University, Zhenjiang 212013, China*

*^77^ Faculty of Sciences and Techniques, Errachidia, Moulay Ismail University of Meknes, Meknes 52000, Morocco*

*^78^ Department of Pharmacology and Physiology, Faculty of Medicine, University of Montreal, Montreal, QC, H3C 3J7, Canada*

*^79^ Research Institute for Future Food, The Hong Kong Polytechnic University, Hung Hom, Hong Kong SAR*

*^80^ Department of Food Science and Nutrition, The Hong Kong Polytechnic University, Hung Hom, Hong Kong SAR*

*^81^ Laboratory of Natural Products and Medicinal Chemistry (LNPMC), Center for Global Health Research, Saveetha Medical College and Hospital, Saveetha Institute of Medical and Technical Sciences (SIMATS), Thandalam, Chennai 602105, India*

^*^Correspondence:

Ren-You Gan ([renyou.gan@polyu.edu.hk](mailto:renyou.gan@polyu.edu.hk))

Ahmed Fatimi ([a.fatimi@usms.ma](mailto:a.fatimi@usms.ma))

Atanas G. Atanasov ([atanas.atanasov@lbg.ac.at](mailto:atanas.atanasov@lbg.ac.at))

Supplementary Figures and Tables


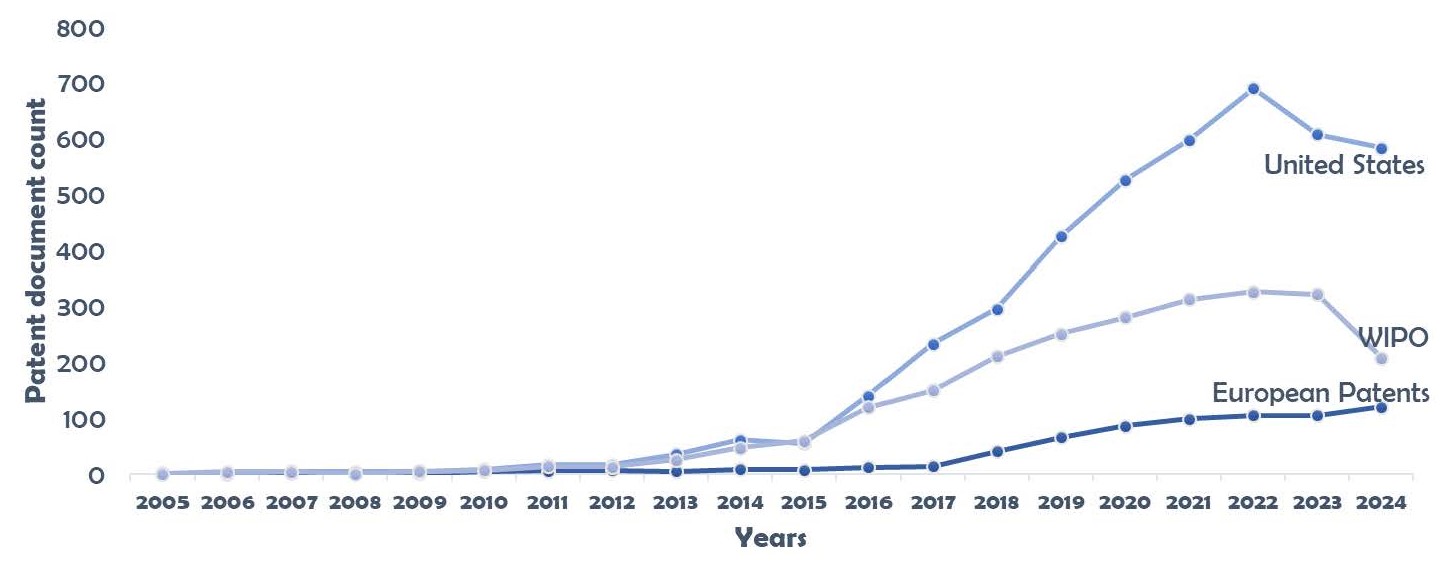


**Figure S1** Dysbiosis patent documents publication over time in the three leading jurisdictions. The trends highlight the growing interest in dysbiosis-related innovations, particularly in the United States, which has consistently led in patent filings. The recent decline observed in 2023-2024 may indicate market saturation, shifts in research focus, or delays in patent processing. WIPO, World Intellectual Property Organization.

**Table S1** Selection of 10 relevant dysbiosis patent documents.
